# Diurnal Human Activity and Introduced Species Affect Occurrence of Carnivores in a Human-Dominated Landscape

**DOI:** 10.1371/journal.pone.0137854

**Published:** 2015-09-14

**Authors:** Dario Moreira-Arce, Pablo M. Vergara, Stan Boutin

**Affiliations:** 1 Department of Biological Sciences, University of Alberta, Edmonton, Alberta, Canada; 2 Laboratorio de Ecología y Conservación, Departamento de Gestión Agraria, Universidad de Santiago de Chile, Santiago, Chile; Instituto de Higiene e Medicina Tropical, PORTUGAL

## Abstract

Diurnal human activity and domestic dogs in agro-forestry mosaics should theoretically modify the diurnal habitat use patterns of native carnivores, with these effects being scale-dependent. We combined intensive camera trapping data with Bayesian occurrence probability models to evaluate both diurnal and nocturnal patterns of space use by carnivores in a mosaic of land-use types in southern Chile. A total of eight carnivores species were recorded, including human-introduced dogs. During the day the most frequently detected species were the culpeo fox and the cougar. Conversely, during the night, the kodkod and chilla fox were the most detected species. The best supported models showed that native carnivores responded differently to landscape attributes and dogs depending on both the time of day as well as the spatial scale of landscape attributes. The positive effect of native forest cover at 250m and 500 m radius buffers was stronger during the night for the Darwin's fox and cougar. Road density at 250m scale negatively affected the diurnal occurrence of Darwin´s fox, whereas at 500m scale roads had a stronger negative effect on the diurnal occurrence of Darwin´s foxes and cougars. A positive effect of road density on dog occurrence was evidenced during both night and day. Patch size had a positive effect on cougar occurrence during night whereas it affected negatively the occurrence of culpeo foxes and skunks during day. Dog occurrence had a negative effect on Darwin's fox occurrence during day-time and night-time, whereas its negative effect on the occurrence of cougar was evidenced only during day-time. Carnivore occurrences were not influenced by the proximity to a conservation area. Our results provided support for the hypothesis that diurnal changes to carnivore occurrence were associated with human and dog activity. Landscape planning in our study area should be focused in reducing both the levels of diurnal human activity in native forest remnants and the dispersion rates of dogs into these habitats.

## Introduction

Spatial distribution and habitat use of wildlife is a dynamic process involving species-specific responses at differing spatial and temporal scales [1–3]. However, understanding habitat use by highly mobile species such as carnivores may be complex. These species exhibit marked diurnal fluctuations in a variety of activities such as movement, feeding, resting, hiding, vigilance, defending territory and mating [4–6].

Carnivores, as well as other terrestrial predators living in human-modified landscapes, face not only diurnal variation in prey availability (e.g.,[7]), but also in the risk of contact with humans or introduced carnivores, such as domestic or free-ranging dogs (hereafter referred to as "dogs") (*Canis familiaris*) [8–10]. However, carnivore studies are usually based on the premise that anthropogenic landscape-scale processes that influence the persistence of carnivore populations are invariant over time, at least in the short-term [11]. The replacement, loss and fragmentation of native habitats tend to occur on relatively broad time scales, such as years, decades or even centuries. However, the resulting land-use mosaics are characterized by diurnal heterogeneity in human activities across the landscape [8]. Therefore, the assessment of human disturbances at the landscape-scale requires consideration of the diurnal responses of carnivores to varying anthropogenic activity, including the presence of canids such as dogs that affect carnivore behaviour [10], [12].

Carnivores might exhibit changes in diurnal habitat-use patterns emerging from multiple ecological processes. For example, the use of habitat by carnivores, such as small but suitable forest patches, may be more intense when humans are less active, thus increasing the levels of intra and interspecific interactions into these remnants [13–16]). In addition, animals may explore distant habitat patches during some periods of the day or night due to human-induced habitat loss and transformation. For example, animals may use movement corridors as a means to reduce ecological dispersal costs [17–19] or avoid human-made structures such as roads during peak hours of traffic (e.g., [20], [21]). Carnivores may also exhibit behavioural changes when approaching habitats influenced by human activities. For instance, during nocturnal forays in human-dominated areas, some carnivores are more cryptic while displaying an opportunistic foraging behavior [22],[13]. Furthermore, in landscapes containing conservation areas surrounded by human land uses (e.g., forest plantations, agricultural lands), the distance over which carnivores carry out incursions may increase as human activity decreases [23]. Lastly, dogs can exclude native carnivores from using high-quality patches [10], and this effect may be more evident during the hours when dogs are more active within these habitats (e.g.,[24]). However, native carnivores may reduce encounter rates with dogs by avoiding using landscape features in the hours dogs are more active [12].

We studied diurnal changes in space use of carnivores by evaluating their occurrence patterns during day and night separately in a human-dominated land-use mosaic of southern Chile. The study mosaic harbors a diverse carnivore guild, including the threatened Darwin's fox (*Pseudalopex fulvipes*) and kodkod cat (*Leopardus guigna*) [25], [26]. Carnivores living in southern Chile have been exposed to human pressure over the last century that has led to a dramatic replacement of native forest into monocultures of exotic trees [27], [28]. Previous studies suggest that some carnivores inhabiting land-use mosaics of southern Chile may be negatively affected by forest plantations, whereas other species would positively respond to these human-created habitats (e.g., [29], [30]). Interactions between carnivores and domestic dogs tend to increase in agricultural and forestry land uses [30], [31]. Agriculture and forestry practices are predominantly carried out during daylight hours across the landscape. Therein, native carnivores would have prolonged exposure to humans and dogs during these hours.

We used a novel spatial Bayesian model to test the hypothesis that carnivores modify their space use patterns from day to night in order to reduce the probability of encountering or being detected by humans and dogs. We assumed that the diurnal period was correlated with high human activity and dog presence as previously documented in natural and more anthropized areas [12], and specifically predicted i) the positive effect of patch size and native forest cover on the occurrence of native carnivores should be more pronounced during the day-time, when human activity is more intense, ii) the occurrence of native carnivores should decrease as road density increases in the landscape, with this effect being stronger during the day, iii) the positive relationship between proximity to a conservation area and the occurrence of native carnivores should be more pronounced during the day-time when levels of human activity around conservation areas increase, and iv) the negative effect of dogs on the occurrence of native carnivores should be more intense during day, when dogs move along roads and explore habitats away from dwelling areas.

## Materials and Methods

### Ethics Statement

Chilean National Forest Service (CONAF) granted permission to conduct camera-trapping surveys within Nahuelbuta National Park. Arauco and Mininco Forestry companies granted permission to conduct camera-trapping surveys on private lands. No Institutional Animal Care and Use Committee (IACUC) or equivalent animal ethics committee was required because we used camera-traps for this study not involving direct contact or interaction with the animals. Camera-trap sampling within National Park agreed with Chilean Forestry Service procedures.

### Study Area

**Fig 1:**
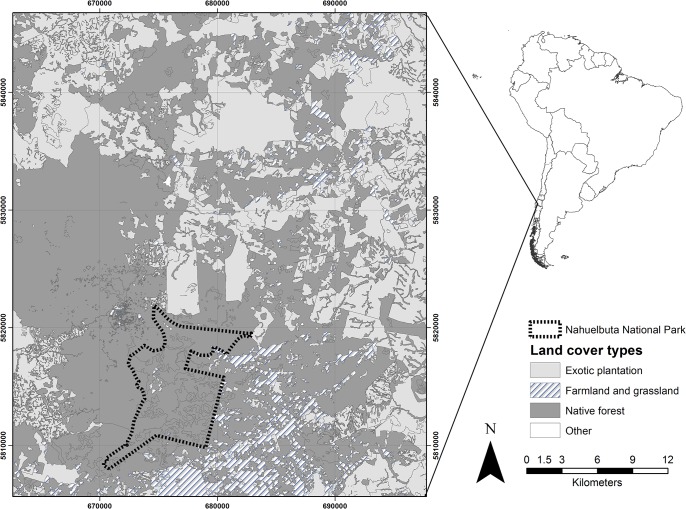
Map of the study area characterized by a human-dominated land-use mosaic surrounding the Nabuelbuta National Park.

Our study area encompassed ca. 1,960km^2^ and was located within the Nahuelbuta Mountain Area (NMA; Fig 1). Climate in this region is warm-temperate with 1,500–3,000 mm of rain concentrated mainly during the austral fall and winter, with frequent snow fall during the winter at high elevations. Elevation ranges from 400 to 1100 m with rugged topography containing numerous ravines and ridges. Historically this region was covered by continuous forest composed by evergreen trees such as *Araucaria araucana*, *Eucryphia cordifolia*, *Aextoxicon punctatum* and *Laureliopsis philippiana* and a mixture of *Nothofagus* species [32]. Currently, the landscape is a mosaic of human-created lands surrounding the Nahuelbuta National Park (NNP), composed of a combination of exotic forest plantation stands of Monterrey pine (*Pinus radiata*) and *Eucalyptus* spp., and remnants of native forest in different successional stages (Fig 1).

### Carnivore species and habitat preferences

A total of seven native carnivores were expected to occur in the study landscape [33], ranging in size from the lesser grison (*Galictis cuja*), Molina's hog-nosed skunks (*Conepatus chinga*) and kodkod to cougar (*Puma concolor*). The habitat-specialist species include the endemic and critically endangered Darwin’s fox [25], [34], a forest-specialist fox species mainly occurring in less disturbed forest of *Araucaria araucana* [34]. Similarly, kodkod cat mainly occurs in continuous and fragmented native forest with denser understory [29]. Habitat-generalist species comprise chilla fox (*Pseudalopex griseus*) and culpeo fox (*Pseudalopex culpaeus*) that occur in a variety of habitats, including native forest and shrub, exotic plantation and grasslands [29], [30]. Lesser grison has been described using native forest [35] and exotic plantations [36]. Studies conducted in Patagonia have shown Molina's hog-nosed skunks (*Conepatus chinga*) selecting open vegetation when active and shrub-forest when resting [37]. Cougars have been recorded using a variety of habitat types, including old-growth native forest, second-growth forest with low canopy cover and grasslands [38], [39]. To our knowledge, no other carnivore species has been documented to occur in our study area.

### Camera-trap sampling

The presence of carnivores was monitored using intensive camera-trap surveys on a sampled areas ca. 1,200km^2^, between November 2011 and December 2012. Camera traps provide a non-invasive method for providing data for estimating spatio-temporal patterns of carnivore occurrence because they record the time and location at which each individual is "trapped" [40]. A total of 210 sites were sampled with passive infrared-triggered camera traps (Reconyx PC900 Holmen, Wisconsin and Bushnell Trophy Camera, Bushnell Corporation, Overland Park, Kansas, USA) mounted on trees ca. 50–60 cm above the ground, and baited with a lure (commercial fox urine, Predator Pee, Maine, USA) 3–4 m away from the camera. We estimated the percentage of sampled points where each species was recorded, which represents an uncorrected or ‘‘naïve” estimate of carnivore occurrence across the entire study area. At each sampling site, photos of the same species taken during a 24hr period were considered as the same detection event to avoid false counts emerging from temporal dependence. Although the study was conducted for approximately one year, each sampling site was surveyed, on average, for 37±12 days during one season only (i.e., during either the breeding or no-breeding season). Once the survey period for a camera was completed, it was moved to a different site, completing a total of 9450 camera-days for the whole study area. Day was defined as 1 h before sunrise until 1h after sunset. Conversely, night was defined as 1h after sunset to 1h before sunrise. The daily sunlight and sunset times were obtained from a sun/moon calculator using the GPS coordinates of the center of the study landscape as reference.

Sampling sites were allocated randomly in the study area based on a habitat-stratified design. However, we maintained a minimum distance of 500m between cameras to promote the spatial independence among detections. We classified the dominant habitat types as being native forest, exotic forest plantations or open farmlands-grasslands by using a 1:250,000 scale landcover GIS database developed by the Chilean Forestry Service and Environment Ministry of Chile and satellite images available in Google Earth (earth.google.com).

### Model covariates

To test the predictions described above, we quantified landscape and habitat covariates associated with each camera station that could affect carnivore detection and occurrence probabilities including: road density, native forest cover, patch size and proximity to conservation area, as well as the occurrence probability of dogs during day and night (Table 1). Landscape attributes were quantified at two spatial scales by creating 250m and 500m-buffer areas around each station in order to include scale-specific effects of landscape attributes on the occurrence of carnivores (e.g., [41], [42]). Spatial data analyses were conducted using ArcMap10.1 (ESRI, CA, USA). For the posterior analyses described below, non-categorical covariates were normalized, when possible, with log transformation, as well as standardized to have a mean 0 and standard deviation 1 to improve model convergence. Using Spearman correlation and the variance inflation factor of all covariates we did not find a strong collinearity between non-categorical covariates. Indeed the absolute values of correlation coefficients between all covariates were < 0.62, while their variance inflation factors were < 3.1 (S1 Table).

**Table 1:** Description of the covariates used in the hierarchical occurrence probability and detection probability models. Model covariates include landscape attributes that were measured at different spatial scales (plot, 250m-radius buffer and 500m-radius buffer).

Code	Variable description
**Occurrence probability model**	
Elv	Elevation (meter above level sea)
Prk	Distance between each camera station and Nahuelbuta National Park border
NF.plot	Binary variable indicating if camera-station was set in native forest or others vegetation type (mainly exotic plantation)
NF250	Native forest cover within 250m-radius buffer area around camera-station
NF500	Native forest cover within 500m-radius buffer area around camera-station
Rd250	Road density, measured as total m of road (paved and dirty road) within an area (km^2^) of 250m-radius buffer area around camera-station
Rd500	Road density, measured as total m of road (paved and dirt road) within an area (km^2^) of 500m-radius buffer area around camera-station
Pch250	Mean patch size (ha) of native forest within 250m-radius buffer area around camera-station
Pch500	Mean patch size (ha) of native forest within 500m-radius buffer area around camera-station
Dog	Occurrence probability (*ψ*_*Dij*_) at camera-station as estimated from model including the effect of landscape and habitat covariates on dogs' detection probability
**Detection probability model**	
Season	Proportion of camera-days sampled in the Austral spring-summer
Und	Percentage of understory vegetation within the detection range of each camera

We considered the carnivore detection probability as a variable being affected by the site-level factors that influenced the chance of, and time when, individuals entered the camera's detection zone. We estimated the cover of understory measured within 10 m in front of each camera station. Because the detection zone of cameras may be blocked by vegetation, we measured understory vegetation blocking the camera's field of view by using a 1x1 m checkerboard (modified from [43]). We included the camera station as a random variable in models to control for the effects of other unobserved variables at the site-level. During their breeding season, density and movement of carnivores can increase, making individuals more detectable in some areas[44] [45]. To account for seasonal changes in detectability of carnivores we included the proportion of camera-days sampled during the Austral spring-summer, corresponding to the breeding season for most of these species.

### Modeling framework

The statistical approach used to assess the space use pattern of the carnivores detected during camera trap surveys can be briefly described as follows: First, we specified a hierarchical single-species/single-season model for the occurrence probability of carnivores (*ψ*) detected during our camera trap surveys. We applied the model described by MacKenzie et al. [46], and further used by Burton et al. [47] for a multi-species assessment, but modified to evaluate the occurrence probability separately during day and night for each carnivore species. Second, to account for imperfect detection on uncorrected estimates of occurrence process, our modelling approach explicitly included the probability of detection (*p*) as a latent (unobserved) variable dependent on environmental covariates [46]- [48]. Third, since species occurrence during day and night are not mutually exclusive events, we used predictions from the hierarchical occurrence model to obtain overall probability of occurrence.

We assumed that presence or absence of a carnivore species at the site *i* = 1,2, .N = 210, during the time period *j* (*j* = 1 if day and *j* = 2 if night) is an Bernoulli distributed latent variable, *z*_*ij*_∼*Bern*(*ψ*_*ij*_), where *z*_*ij*_ = 1 if the species is present and *z*_*ij*_ = 0 if the species is absent, while *ψ*_ij_ is the probability that the species occurs at site *i* during the time period *j*. We modeled observed detections, *y*_i,j,_ as *y*_*ij*_ ∼ *Bern*(*z*_*ij*_ *p*_*ij*_) for *k*_j_ independent trials, where *p*_*ij*_ is the probability of detecting species at site *i* during the time period *j* if it is present, and *k*_*j*_ is the number of trap days at site *j* as bivariate logit-normal random variables. Occurrence probability adjusted for imperfect detectability was modelled as:

$$logit\left(\psi_{ij}\right)=\alpha_{1j}+\beta_{j}\;X_{i}+\gamma_{j}\psi_{Dij}+U_{i}+d_{j}$$ Eq.(1)

where *α*_1*j*_ is an intercept parameter. The occurrence probability function given in Eq (1) includes a vector of the time-dependent coefficients, *β*_*j*_, for day and night periods (*j* = 1 and *j* = 2, respectively), associated with a vector of time-independent covariates at site *i* (*X*_*i*_). These covariates were habitat and landscape attributes influenced by human disturbances at different spatial-scales (Table 1). We included elevation as an additional covariate because the pronounced altitudinal gradient in the study landscape could affect carnivore occurrence due to possible altitudinal gradients in prey abundance and human activity [49]. For each carnivore species, with the exception of dog, the time-dependent coefficient *γ*_*j*_ (Eq 1) represents the probability of a dog being present (*ψ*_*Dij*_) at camera *i* during day and night. We estimated the occurrence probability of dogs, *ψ*_*Dij*_, using a detection model including effects of environmental factors, as explained below in Eq (2), but also by using an occurrence probability function without covariates for not including fixed-effects again in the function (S2 Table). Parameter *d*_*j*_ in Eq (1) is a random effect for day and night, separately, since the assumption of temporal independence of errors was not supported by observations. Parameter *d*_*j*_ was drawn from a bivariate normal distribution d∼N(μ,Σ) whose correlation matrix, *Σ*, provided the coefficient *ρ* representing the correlation between the probability of occurrence estimated during both day and night.

Detection probability, *p*_*ij*_, at site *i* during the time period *j* was estimated by using the Equation:

$$logit(p_{ij})=\alpha_{2j}+\delta_{j}\;X_{i}+S_{i}$$ Eq.(2)

which includes an intercept parameter, *α*_2*j*_, as well as a time-dependent coefficient vector, *δ*_*j*_, representing the factors affecting detection probability (i.e., season and understory vegetation; see Model covariates section for details) at site *i*, *X*_*i*_ (Table 1), and a spatially unstructured random effect, *S*_*i*_, for each site.

A correlation between the occurrence and detection probabilities is probable because an increase of animal activity within a particular area may become individuals more detectable by cameras set within that area [43], [44], [47]. Thus, to account for the positive association between occurrence and detection probabilities, we modeled *ψ* and *p* as bivariate logit-normal random variables. The logit(*ψ*_*ij*_) and logit(*p*_*ij*_) values were combined into the two-dimensional vector *G*_*j*_, such that $G_{j}∼N(\mu_{j},\Sigma )$, where *Σ* is a covariance matrix and *μ*_*j*_ a mean vector that contains the occurrence, *ψ*_*ij*_ and detectability, *p*_*ij*_ probabilities.

In order to include temporal dependencies in occurrence and detection processes, we specified the same hyper-parameters (i.e., parameters of prior distributions of model parameters) for each time-dependent coefficient (*β* and *δ*) representing the effect of the same covariate but at different time period (day or night). Our model assumed that the probability of daily and nightly occurrences at site *i* are correlated random variables. The occurrence probability during the overall 24-hour day was estimated as:

$$\psi_{i}=1-\left[\left(1-\psi_{i1}\right)\left(1-\psi_{i2}\right)\right]$$ Eq.(3)

We controlled for spatial errors associated with the local neighborhood dependencies of camera stations in the occurrence probability function, Eq (1), by including a spatial term for each site *i*, *U*_*i*_, which was drawn from a Gaussian conditional autoregressive (CAR) distribution. The CAR approach assumes a set of area-specific spatially correlated Gaussian random effects [50]. Using Voronoi tessellation applied to the coordinates of camera stations, we subdivided the study landscape into non-overlapping areas (Voronoi polygons), each representing an “influence” area associated to each camera station. The elements of the adjacency matrix used for specifying the CAR function were defined as those Voronoi polygons that shared a boundary (e.g., [51]).

We selected models using the posterior probability of all possible combinations of fixed-effects coefficient (*β* and *δ*), including a set of 2^20^ candidate models. Model ranking based on their posterior probabilities provides a suitable selection procedure for complex hierarchical models with latent variables, such as our hierarchical occurrence probability model [52]. Models with posterior probabilities >0.05 were considered to be the suitable supported models. Posterior probabilities were calculated by fitting inclusion parameters, *w*_c_, to each fixed effect coefficient, where *C* is the complete set of fixed effects. The inclusion parameter gives the probability that a particular covariate is included in the “best” model. Inclusion parameters were assumed to be Bernoulli distributed and specified with uninformative prior probability parameter of 0.5. From Markov Chain Monte Carlo (MCMC) samples we estimated the posterior probability of each model by calculating the proportion of times each combination of fixed effects appeared in the posterior sample (i.e., when w_c_ = 1 for all model coefficients).We estimated model-averaged coefficients from posterior samples by averaging values where the corresponding w_c_ = 1 [51]. The importance of each fixed effect was evaluated from the Bayesian credible intervals of the posterior distribution of coefficients. We only interpreted coefficients whose 95% credible intervals did not overlap zero.

Models were run using WinBUGSv. 1.4 [53], which was remotely called from R v. 3.2.0 (R Development Core Team 2014) by using the R2WinBUGS package. Posterior distributions were based on five MCMC iterations, each with 40,000 iterations, discarding the first 10,000 iterations and thinning by 5.We used vague non-informative prior distributions for all model parameters. We assessed convergence by visually examining trace and density plots of MCMC iterations as well as by estimating the Potential Scale Reduction factor [54].

## Results

### Occurrence patterns

Eight carnivore species were recorded during camera-trapping surveys, with occurrence rates (naïve estimate of carnivore occurrence) differing between day and night (Table 2 and S3 Table). The culpeo fox, followed by the cougar, were the most frequently recorded species during day, present at >40% of the sampled sites (Table 2). During the night, the more frequently recorded species were the kodkod and chilla fox, with both species being detected at >30% of the sampled sites (Table 2). Diurnal variation in estimated mean of *ψ* was more pronounced for the culpeo fox, for which estimated mean of *ψ* during day was three times larger than during night (Table 2). The dog, cougar and grison had higher estimated mean of *ψ* values during the day than night (7%–20% higher), whereas the Darwin's fox, chilla fox and kodkod had an estimated mean of *ψ* values higher during the night (6% -33% higher). The kodkod and skunk exhibited the largest correlation between day and night estimated mean of *ψ* (*ρ≤* 0.64 for all species), while the culpeo fox was the species with the lowest correlation between day and night estimated mean of *ψ* (Table 2). The culpeo fox, chilla fox, cougar and kodkod exhibited the highest model estimates of overall occurrence probabilities *ψ*_*overall*_≥ 0.7 (Table 2).

**Table 2:** Carnivore species detected during the camera trap survey in Nahuelbuta Mountain Area in southern Chile. For both day and night, the following estimates are reported: The percentage of sampling sites where at least one detection occurred (Detections (%)), the model-averaged estimates (means and SDs from posterior probability distribution of estimates) of occurrence probability (*ψ*), detection probability (*p*) as well as the overall occurrence probability (*ψ*_*overall*_) and the correlation (*ρ*) between day and night.

	Day			Night			Correlation	Overall occurrence
Species	Detections (%)	*ψ* (SD)	*p* (SD)	Detections (%)	*ψ* (SD)	*p* (SD)	ρ (SD)	*ψ*_*overall*_ (SD)
Culpeo fox	0.71	0.80 (0.11)	0.80 (0.02)	0.18	0.24 (0.08)	0.60 (0.07)	0.08 (0.03)	0.83 (0.13)
Chilla fox	0.3	0.65 (0.09)	0.40 (0.05)	0.38	0.77 (0.06)	0.44 (0.04)	0.61 (0.14)	0.91 (0.13)
Kodkod cat	0.33	0.49 (0.1)	0.43 (0.09)	0.37	0.52 (0.1)	0.67 (0.08)	0.64 (0.02)	0.75 (0.15)
Dog	0.34	0.46 (0.13)	0.54 (0.12)	0.21	0.38 (0.18)	0.43 (0.09)	0.58 (0.18)	0.66 (0.19)
Cougar	0.4	0.55 (0.13)	0.58 (0.04)	0.32	0.51 (0.13)	0.58 (0.04)	0.47 (0.32)	0.78 (0.21)
Lesser grison	0.12	0.34 (0.13)	0.27 (0.03)	0.16	0.31 (0.17)	0.43 (0.03)	0.4 (0.03)	0.54 (0.18)
Darwin's fox	0.09	0.14 (0.04)	0.54 (0.13)	0.11	0.21 (0.05)	0.48 (0.17)	0.63 (0.05)	0.32 (0.1)
Skunk	0.17	0.25 (0.08)	0.30 (0.17)	0.13	0.25 (0.06)	0.47 (0.19)	0.64 (0.1)	0.43 (0.14)

#### Patch size and forest cover (prediction 1)

The prediction that the positive effect of patch size and native forest cover on the occurrence of native carnivores should be more pronounced during the day-time was partially supported by results. As explained in details below some carnivores such as chilla fox, skunk and the habitat specialists Darwin's fox and kodkokd positively responded to forest cover. However, this response was scale-dependent and sometimes more accentuated during night. Similarly, the positive effect of patch size was stronger during night for cougars whereas its negative effect was stronger during day for culpeo foxes and skunks.

**Fig 2:**
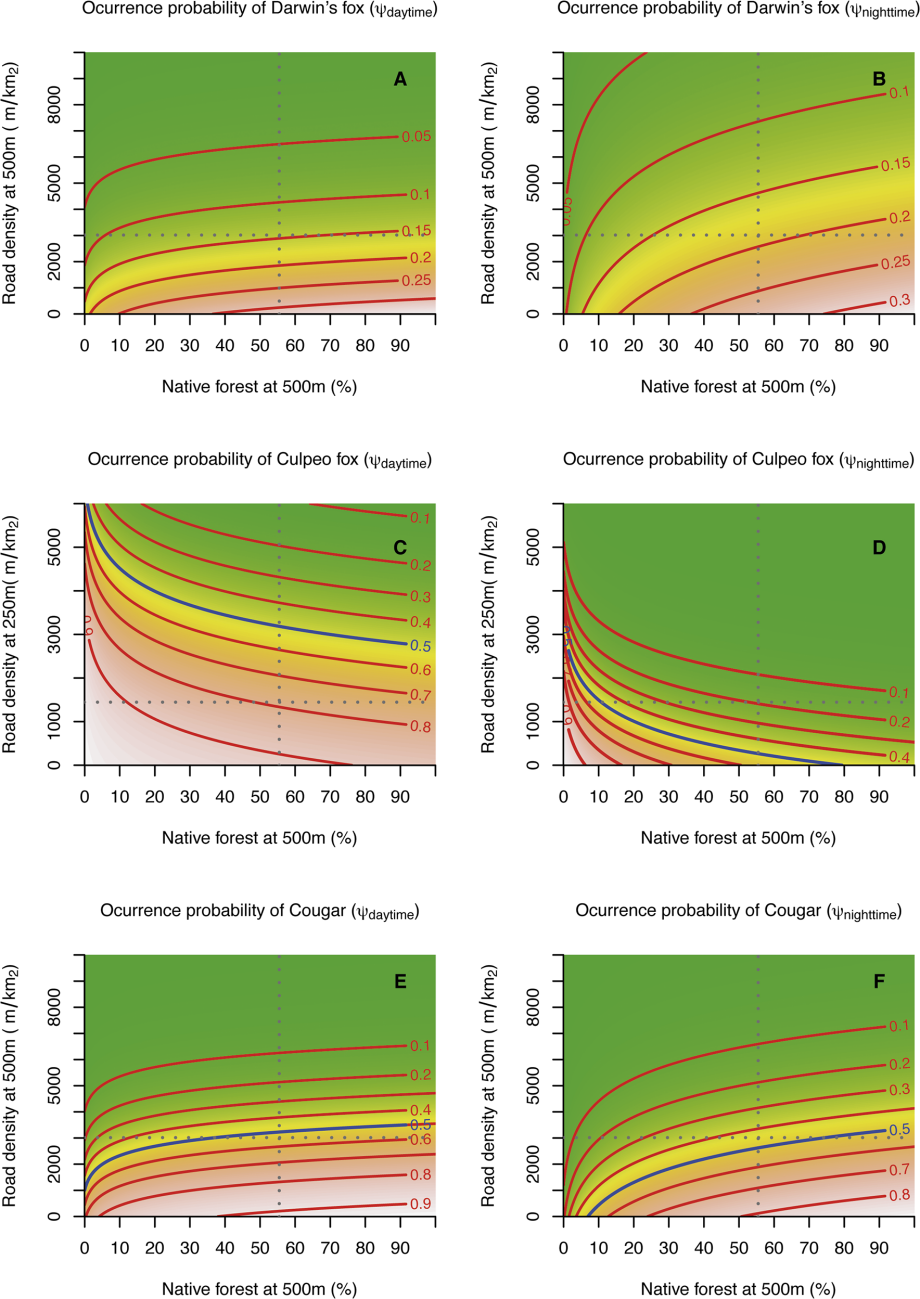
Contour plots showing model-predicted occurrence probabilities (*ψ*) of Darwin’s fox (A and B), culpeo fox (C and D) and cougar (E and F) as function of two landscape—scale covariates: road density at two different scales (250m radius buffer and 500m radius buffer) and the amount (%) of native forest at 500m. Red isolines indicate combinations of the two covariates predicting a particular (*ψ*) level (with the blue isocline showing *ψ* = 0.5). Vertical and horizontal dashed lines indicate the mean value of the covariate, as measured in the study landscape.

The cover of native forest measured at the camera-station (NF.plot) influenced the occurrence probability of all native carnivores (Table 3). However, for some species, such as the chilla fox, skunk and Darwin's fox, this positive effect was only included in the best supported models during the day (Table 3). We also found a positive effect of native forest at the camera-station on the occurrence probability of kodkod, however, this effect was stronger during day (Table 4). In contrast, for the culpeo fox the positive effect of native forest cover at the camera-station was stronger during the night than day-time, as shown by differences in nocturnal and diurnal coefficients of *ψ* (Table 4). The analysis of data from the native forest plots with the 500m radius buffer revealed a temporal effect on the *ψ* of carnivores. Indeed, native forest at the 500m radius buffer had a positive and stronger effect on Darwin's fox and cougar occurrence probability during the night than during the day (Fig 2), whereas a positive effect of this covariate was only included in the top-ranked occurrence probability models of chilla fox during the night (Table 3). In contrast, native forest at 500m radius buffer had a negative effect on the *ψ* of culpeo fox, but that effect was ca. 43% stronger during the night than the day (Table 4, Fig 2). Even though the occurrence probabilities of lesser grison and skunk were positively affected by native forest at at 250m radius buffer, we did not detected a differential effect during day or night.

**Table 3:** Posterior model probabilities for the set of best-supported candidate models (i.e., with posterior probability >0.05) for the occurrence probability (*ψ*) and detection probabilities (*p*) of carnivores in Nahuelbuta Mountain Area in southern Chile.

Species	Day	Night	Posterior probability
**Kodkod cat**	ψ(NF.plot) *p*(Season)	ψ(NF.plot) *p*(Season)	0.163
** **	ψ(Elv) *p*(.)	ψ(Elv) *p*(.)	0.1
** **	ψ(.) *p*(.)	ψ(NF.plot) *p*(.)	0.085
** **	ψ(.) *p*(Season)	ψ(.) *p*(Season)	0.061
** **	ψ(.) *p*(Season)	ψ(.) *p*(.)	0.061
**Chilla fox**	ψ(NF.plot) *p*(Season)	ψ(Dog) p(Season)	0.173
** **	ψ(NF.plot) *p*(Season)	ψ(NF500) *p(*Season)	0.055
** **	ψ(NF.plot+Dog) *p*(Season)	ψ(Pch500) *p*(Season)	0.052
** **	ψ(NF.plot) *p*(.)	ψ(.) p(Season)	0.051
**Lesser grison**	ψ(NF.plot+NF250) *p*(Season)	ψ(NF250) *p* (.)	0.148
** **	ψ(NF.plot+Prk) *p*(Season)	ψ(Prk) p (.)	0.111
** **	ψ(NF.plot+NF250+Dog) *p*(Season)	ψ(Elv+NF250+Dog) *p*(.)	0.074
** **	ψ(NF.plot+NF250) *p*(Season)	ψ(NF.plot+NF250) *p* (.)	0.074
** **	ψ(NF.plot) *p*(Season)	ψ(.) p (.)	0.073
**Culpeo fox**	ψ(NF.plot+Elv+NF500+Pch500+Dog) *p*(Season)	ψ(NF250+Rd250+Pch250) *p*(Season)	0.171
** **	ψ(Pch500+Dog) *p*(Season)	ψ(Elv+Rd250) *p*(Season)	0.072
** **	ψ(NF.plot+NF500+Dog) *p*(Season)	ψ(NF.plot+NF500) *p*(Season)	0.053
** **	ψ(Elv+Rd250+Pch500) *p*(Season)	ψ(Elv+Dog) *p*(.)	0.052
**Darwin's fox**	ψ(NF.plot+Rd250+Dog) *p*(.)	ψ(NF500+Dog) *p*(Und)	0.258
** **	ψ(NF.plot+Elv+NF500) *p*(.)	ψ(NF500) *p(*Season)	0.065
** **	ψ(NF.plot+Elv+Rd250+Rd500+Dog) *p*(Season)	ψ(NF500+Rd500+Dog) *p*(Season+Und)	0.055
**Dog**	ψ(Rd500) *p*(Season)	ψ(Rd250) *p*(Season)	0.229
** **	ψ(NF500) *p*(.)	ψ(NF500) *p*(.)	0.2
** **	ψ(.) *p*(.)	ψ(.) p(Season)	0.089
**Cougar**	ψ(NF.plot+NF500+Rd500+Dog) *p*(.)	ψ(NF.plot+NF500+Rd500) *p*(.)	0.112
** **	ψ(NF.plot+NF500+Dog) *p*(.)	ψ(NF.plot+NF250+NF500+Rd500) *p*(.)	0.106
** **	ψ(Elv+Dog) *p*(Und)	ψ(NF.plot+NF250+Pch500) *p*(Und)	0.062
** **	ψ(NF.plot+NF500+Rd500) *p*(.)	ψ(NF250+NF500+Rd250+Rd500+Pch500) *p*(.)	0.057
** **	ψ(Elv) *p*(.)	ψ(NF.plot+Pch500) *p*(.)	0.057
** **	ψ(.) *p*(.)	ψ(NF.plot+NF250+NF500+Rd500+Pch500) *p*(.)	0.053
**Skunk**	ψ(Elv+NF250+Rd250) *p*(Und)	ψ(NF.plot+Elv+NF250) *p*(Season)	0.135
** **	ψ(Elv+NF250+Pch500+Rd250+Dog) *p*(Season+Und)	ψ(NF.plot+Elv+NF250+Rd250) *p*(.)	0.081
** **	ψ(.) *p*(Season)	ψ(.) *p*(.)	0.051

**Table 4:** Posterior model-averaged coefficients, standard errors (SE), 95% credible interval (CI) and inclusion probability for covariates included in the best supported models (see Table 3) which are expected to influence diurnal and nocturnal occurrence (*ψ*) and detection (*p*) probabilities of carnivores in Nahuelbuta Mountain Range in southern Chile. (-) Covariate not included in the best-supported candidate models.

		Day			Night		
Species	Covariate	Mean (SE)	95% CI	Inclusion probability	Mean (SE)	95% CI	Inclusion probability
**Kodkod**	NF.plot	2.17 (0.01)	2.09, 2.16	0.64	1.90 (0.10)	2.17, 2.16	0.56
** **	Elv	-0.35 (0.02)	-0.39, -0.32	0.58	-0.34 (0.01)	-0.38, -0.31	0.52
** **	Season	-0.84 (0.12)	-0.86, -0.82	0.59	-0.83 (0.12)	-0.86, 0.81	0.41
**Chilla fox**	NF.plot	0.53 (0.02)	0.49, 0.57	0.6	-	-	-
** **	Pch500	0.15 (0.014)	0.12, 0.18	0.69	0.10 (0.015)	0.07, 0.13	0.59
** **	NF500	-	-	-	0.19 (0.018)	0.15, 0.22	0.62
** **	Dog	0.79 (0.09)	0.61, 0.97	0.59	0.8 (0.08)	0.63, 0.98	0.82
** **	Season	1.14 (0.001)	1.14, 1.18	0.53	1.2 (0.001)	1.18. 1.22	0.58
**Lesser grison**	NF.plot	3.26 (0.20)	2.88, 3.63	0.87	3.24 (0.19)	2.86, 3.62	0.68
** **	NF250	0.90 (0.13)	0.64, 1.15	0.61	0.91 (0.13)	0.64, 1.16	0.7
** **	Prk	-0.04 (0.15)	-0.35, 0.26	0.12	-0.04 (0.15)	-0.34, 0.27	0.15
** **	Elv	-	-	-	-0.23 (0.17)	-0.57, 0.10	0.12
** **	Dog	0.31 (0.2)	-0.08, 0.70	0.34	0.35 (0.3)	-0.24, 0.94	0.37
** **	Season	1.76 (0.07)	1.62, 1.89	0.84	-	-	-
**Culpeo fox**	NF.plot	2.53 (0.25)	2.02, 3.03	0.55	2.82 (0.26)	2.2, 3.20	0.72
** **	Elv	-0.14 (0.13)	-0.41, 0.12	0.32	-0.14 (0.13)	-0.41, 0.12	0.38
** **	NF500	-0.61 (0.14)	-0.89, -0.32	0.76	-0.87 (0.13)	-1.14, -0.60	0.61
** **	Pch500	-0.50 (0.15)	-0.81, -0.19	0.66	-	-	-
** **	Rd250	-1.4 (0.19)	-1.77, -1.03	0.66	-1.9 (0.18)	-2.25, -1.54	0.75
** **	NF250	-	-	-	-1.43 (0.18)	-1.79, -1.07	0.63
** **	Dog	1.01 (0.17)	0.67, 1.36	0.63	0.90 (0.17)	0.64, 1.33	0.3
** **	Season	-1.26 (0.14)	-1.54, -0.97	0.82	-1.26 (0.14)	-1.53, -0.98	0.64
**Darwin's fox**	NF. Plot	2.44 (0.21)	2.02, 2.86	0.58	-	-	-
** **	Elv	-0.73 (0.15)	-1.04, -0.42	0.54	-	-	-
** **	NF500	0.18 (0.08)	0.02, 0.33	0.29	0.38 (0.10)	0.18, 0.57	0.4
** **	Rd250	-0.72 (0.15)	-1.02, -0.42	0.75	-	-	-
** **	Rd500	-0.94 (0.13)	-1.04, -0.50	0.78	-0.49 (0.17)	-0.74, -0.23	0.65
** **	Dog	-0.27 (0.14)	-0.54, -0.003	0.74	-0.27 (0.13)	-0.54, -0.01	0.88
** **	Season	0.19 (0.15)	-0.12, 0.50	0.17	0.18 (0.16)	-0.13, 0.50	0.29
** **	Und	-0.61 (0.09)	-0.79, -0.43	0.76	-0.64 (0.09)	-0.83, -0.47	0.67
**Dog**	Rd500	0.29 (0.01)	0.26, 0.32	0.82	-	-	-
** **	NF500	-0.26 (0.13)	-0.01, -0.51	0.66	-0.50 (0.20)	-0.11, -0.89	0.72
** **	Rd250	-	-	-	0.10 (0.02)	0.07, 0.12	0.55
** **	Season	-3.94 (0.05)	-4.05, -3.83	0.77	-3.99 (0.05)	-4.09, -3.89	0.78
**Cougar**	NF.plot	3.49 (0.24)	3.00, 3.97	0.7	3.50 (0.24)	3.02, 3.98	0.79
** **	Elv	-0.18 (0.15)	-0.48, 0.13	0.16	-	-	-
** **	NF500	0.44 (0.15)	0.14, 0.74	0.72	0.74 (0.18)	0.38, 1.09	0.86
** **	Rd500	-1.90 (0.06)	-2.01, -1.78	0.72	-1.75 (0.11)	-1.96, -1.53	0.65
** **	NF250	-	-	-	2.03 (0.14)	1.74, 2.31	0.57
** **	Pch500	-	-	-	0.68 (0.16)	0.36, 1.00	0.72
** **	Rd250	-	-	-	0.02 (0.16)	-0.30, 0.34	0.24
** **	Dog	-1.11 (0.16)	-1.42, -0.80	0.78	-	-	-
** **	Und	-0.21 (0.15)	-0.51, 0.09	0.22	-0.22 (0.16)	-0.52, 0.1	0.25
**Skunk**	NF.plot	1.89 (0.18)	1.52, 2.26	0.52	-	-	-
** **	Elv	-0.22 (0.16)	-0.53, 0.09	0.38	-0.24 (0.16)	-0.55, 0.08	0.38
** **	NF250	0.27 (0.15)	-0.03, 0.57	0.29	0.26 (0.15)	-0.04, 0.56	0.27
** **	Rd250	-0.36 (0.14)	-0.63, -0.08	0.65	-0.59 (0.14)	-0.86, -0.30	0.67
** **	Pch500	-0.34 (0.17)	-0.67, -0.01	0.52	-	-	-
** **	Dog	-0.08 (0.15)	-0.37, 0.21	0.49	-	-	-
** **	Season	-2.45 (0.12)	-2.69, -2.21	0.67	-2.46 (0.12)	-2.70, -2.22	0.65
** **	Und	-0.35 (0.14)	-0.62, -0.07	0.58	-	-	-

Occurrence probability was influenced by patch size for four of the eight carnivores, with this effect being mainly found at the 500m scale (Table 3). We detected a positive effect of patch size on the cougar occurrence probability only during night, whereas it negatively affected the *ψ* of culpeo foxes and skunks during day (Table 4). Even though patch size at the 500m scale affected the occurrence probability of chilla fox, this effect was 50% stronger during day than night (Table 4).

#### Road density (prediction 2)

The prediction that the occurrence of native carnivores should decrease during the day as road density increases in the landscape was partially supported by the results. As explained in details below, only the cougar and Darwin's fox responded more strongly, and negatively, to road density during day whereas the habitat generalists culpeo foxes and skunks were negatively affected by roads during night.

Road density influenced the nocturnal and diurnal occurrence probabilities of carnivores at multiple spatial-scales, as indicated by the best supported occurrence models (Table 3). Road density at 250m scale negatively affected the occurrence probability of Darwin's fox only during day (Table 4). Road density at 250m scale more strongly reduced the nocturnal occurrence of culpeo fox and skunk than the diurnal occurrence (Table 4, Fig 2). Conversely, road density at 250m scale positively affected the nocturnal occurrence of dogs (Table 4). At the 500m scale, road density negatively affected both diurnal and nocturnal occurrence probabilities of Darwin's fox and cougar, with this effect being 31% and 27% stronger during day, respectively, as shown by differences between diurnal and nocturnal coefficients (*β*) (Table 4, Fig 2). Road density at 500m scale, however, showed a positive effect of dog occurrence probability during day-time only (Table 4).

#### Proximity to a conservation area (prediction 3)

The prediction that the positive relationship between proximity to a conservation area and the occurrence of native carnivores should be more pronounced during the day-time was not supported by results. Only the best supported occurrence models for the lesser grison included the proximity to a conservation area as a covariate, but the effect was not significant (Tables 3 and 4).

#### Dog occurrence (prediction 4)

The prediction that the negative effect of dogs on the occurrence of native carnivores should be more intense during day was only hardly supported among carnivores. Conversely, dogs negatively affected most of the carnivores, independently from the time throughout the day as indicated by the best supported occurrence models (Table 3); and for some species such as culpeo and chilla foxes, their occurrence probabilities were positively associated with dogs (Table 3). Dogs negatively affected the occurrence of Darwin's fox during day-time and night-time whereas its negative effect on the occurrence of cougar and skunk was evidenced only during day-time (Table 4). In contrast, dogs were positively associated to the occurrence probabilities of chilla fox, culpeo fox and lesser grison during day and night with similar magnitude (Table 4).

## Discussion

Our results support the hypothesis that diurnal changes in space use by carnivores were associated with human and dog activity. These findings expand our understanding of the dynamics of the flexible habitat use by carnivores, which have been previously found to occur on a seasonal or annual basis rather than on diurnal scales, such as shown in this study (see Fig 2). Although previous studies have addressed the temporal occurrence patterns of carnivores (e.g., [55]), shorter-temporal responses such as diurnal occurrence patterns of carnivores across human modified landscapes have been poorly studied. Landscape ecology theory has contributed greatly to our understanding about the ecological effects of land use changes, such as deforestation or land degradation, which typically occur at relatively broader temporal scales [8]. However, human-dominated landscapes are short-term dynamic systems, with human activities being more intensive at different times throughout the day. Thus, results of this study provide new insights for the ecology of threatened carnivore species and their behavioral responses in human-dominated landscapes.

All the study carnivore species, including the threatened Darwin's fox, had relatively high estimates of overall occurrence probability (*ψ*_*overall*_ >0.3; Table 2). However, the carnivores did not exhibit similar occurrence probabilities between day and night, nor did they respond in a consistent manner to changes in human and dog activity. The occurrence patterns of carnivores were larger during either the night or day, depending on both the species and the spatial scale. The best-supported models suggest that the variable effects of landscape attributes on the carnivores' occurrence depend on time the time of day in which the species are more actively searching for prey, as well as are willing to move to, and use, the habitats where prey are available. We confirmed the positive and negative effect of the native forest on previously described forest-specialist and habitat-generalist carnivores, respectively. However, our results also indicate that the habitat effect is time and spatial scale-dependent. Previous studies in Temperate Forest have documented that culpeo, chilla foxes and even cougars exhibit a habitat-generalist behavior, using habitats with intensive land use and disturbance (e.g., forest plantations or agricultural lands)[29],[30],[36], unlike the Darwin's fox and kodkod cat which have been documented to use more undisturbed forest [29],[56]. Moreover, behavior of domestic dogs in semi-natural landscapes of southern Chile has been previously reported [30],[57]. However, habitat specificity of the carnivore species included in this study changed between day and night. As supported by our findings, and discussed in details below, forest-specialized species, like Darwin's foxes and kodkod cats, showed stronger preferences for native forest during night and day, respectively. Similarly, cougars also showed stronger preference for large patches with native forest during night, whereas avoiding areas with larger road densities during day as previously documented in other regions [58]. Conversely, habitat-generalist fox species, such as culpeo fox, strongly avoided native forest but preferently during night in our study area, whereas avoiding large patches of forest during day and areas with higher road density preferently during night. However, for chilla fox, we found that its occurrence probability during day increased with the presence of native forest, suggesting a more habitat-specialist behaviour in our study area during a specific period of the day. The occurrence probabilities of other previously described habitat-generalist species such as skunk and lesser grison increased with the presence of native forest. However for skunk, this effect was scale-dependent and evidenced during day, suggesting a more specialist behavior during this period. These findings are therefore novel in showing that habitat attributes (e.g., native forest cover) can affect the space use patterns of both habitat-generalists and forest-specialists, but that these effects change throughout day.

### Patch size and forest cover (*prediction 1*)

We found that diurnal occurrence of native carnivores were predicted by both native forest availability at different spatial scales and patch size. For Darwin's foxes and cougars, the stronger positive nocturnal effect of the amount of forest area and patch size of native forest at the 500m scale suggests that these species can concentrate their foraging effort in landscapes with more native forest during night. Native forest provides shelter as well as food resources such as small mammals, which are particularly abundant and constitute the major prey type for Darwin's fox [29]. In these landscapes, Darwin's fox, may also face reduced interference-competition from the habitat-generalist culpeo fox and dog who, during night, avoided landscapes with native forest (Table 4 and Fig 2). On the other hand, cougars would benefit from large remnants of native forest due to the southern Pudu (*Pudu puda*), one of their main native prey, occurring frequently in these habitats [57]. The broad space use by carnivores, however, should be understood by considering their short-term patterns of occurrence in landscapes. For example, culpeo foxes and skunks avoided landscapes with large native forest remnants only during day-time. Furthermore, the combination of time of the day and spatial scales at which the effects of landscape attributes become more intense may influence space use of carnivores [59]. For example, the cover of native forest at camera stations increased the occurrence of most native carnivores in our study area. However, the positive effect of native forest at this scale was only detected during day-time for chilla fox, skunk and Darwin's fox, possibly suggesting that these small-sized carnivore species use native forest remnants as a day-time refuge, reducing the probability of encounters with humans or dogs [12]. Similarly, the stronger effect of native forest at camera station scale on kodkod cat during day support this habitat provides shelter for this habitat-specialist felid but would also suggest native may act foraging habitat for this tree-climber species [60]. In addition, although the occurrence of culpeo fox decreased in areas covered by native forest [29], the less pronounced effect of this factor during the day-time (Fig 2) suggests native forest can potentially function as a habitat free of human activities for this predator.

### Road density (*prediction 2*)

Native carnivores, such as cougar and Darwin's fox were less likely to occur in areas with high road density, and this effect was more pronounced during the day, probably as a response to increased traffic levels on the roads during the day-time (Fig 2). Culpeo fox, however, had lower occurrence probabilities in areas with a high road density during night, probably to avoid encounters with dogs which responded positively to roads during night. Although areas with old and partially overgrown road cover may have a positive effect on carnivore activity by providing access to edge habitats where many prey are vulnerable [61], examples of carnivores responding negatively to dense road networks that act as movement barriers or mortality source prevail in ecological literature (e.g. [62], [63], [64]). Diurnal variation in how strongly roads influence carnivores could reflect a changing risk perception, which, in turn, may be triggered by previous encounters with humans, cars and dogs along roads during day-time [12]. Short-term behavioral plasticity, promoted by changing habitat quality and availability or by variable human activity, could be critical for survival of carnivores living in human-dominated land-use mosaics, as those species present in our study region [65].

### Proximity to a conservation area (*prediction 3*)

Contrary to the third prediction, carnivore occurrence did not response to proximity of Nahuelbuta National Park. This finding suggests that there is not a spatial gradient in habitat quality promoting an increased spatial use near the national park. In addition, it is possible that the Nahuelbuta National Park is not large enough to support viable local populations [66]. The role of protected areas on species conservation depends largely on the level of human activity that occurs in the matrix (e.g., agriculture, forestry or housing) surrounding protected lands [67] Our results suggest that unprotected, large, native forest patches located northwest to Nahuelbuta National Park (see Fig 1) play an important role in providing adequate habitat conditions for native carnivores, therefore favouring animal movement within this landscape.

### Dog occurrence (*prediction 4*)

Our hypothesis that the diurnal occurrences of carnivores are influenced by landscape-scale human disturbances can be generalized by effects beyond habitat loss and degradation. Introduced species (e.g., dogs), that can be benefited by these environmental changes, increase the effect of human disturbance on biodiversity [10]. In fact, the occurrence of dogs was largely influenced by road density (positive) and native forest (negative) at different spatial scales, supporting that the detrimental effects of this introduced carnivore on local biodiversity is shaped by human land use. In southern Chile free-ranging dogs have also been suggested to move preferentially through roads and using human-created open areas [30], [57]. In contrast, as shown in this study, cougars, chilla foxes and Darwin's foxes tend to avoid areas with more roads while using large patches of native forest (Table 4 and Fig 2). Therefore, native carnivores respond differentially during day or night to not only the landscape attributes, but also to the diurnal use and movement of introduced carnivores across the landscape. However, dogs were positively associated with chilla fox and culpeo fox. Such a positive association should emerge more from a similarity in habitat preferences rather than from a positive interaction between species (e.g., commensalism or mutualism). Thus, chilla fox and culpeo fox may have increasingly more interactions with dogs than the other carnivore species.

## Concluding remarks

Our results suggest that native carnivores inhabiting this human-dominated landscape, and in particular the threatened Darwin´s fox, occur preferentially in habitats covered by larger amounts of native forest and larger forest patches while displaying diurnal behaviors intended to reduce the encounters with humans and introduced dogs. However, in landscapes experiencing increased forest loss or degradation, carnivores can concentrate into the few patches, thus increasing the levels of spatial overlap among different carnivore species. Future studies addressing the hunting time activity of native carnivores are required to provide a conservation basis for reducing human effects on the foraging success of carnivores. Finally, we stress the need to 1) increase the patch size of native forest remnants; 2) develop an integrated management strategy taking into account large native-forest patches that belong to forestry companies as well as small native forest remnants that belong to smaller landowners; and 3) re-vegetate unused forestry roads and paths and implement dog-free zones to reduce the lethal and non-lethal effects of this exotic carnivore on native fauna.

## Supporting Information

S1 Table Correlation coefficients (top) and variance inflation factors (down) for covariates used in models of carnivore occurrence and detection probability. (DOCX)

S2 Table Posterior model probabilities for the set of best-supported candidate models (i.e., with posterior probability >0.05) for the detection (*p*) probabilities of introduced dog in Nahuelbuta Mountain Area in southern Chile. The occurrence of dogs was estimated from a null model.
(DOCX)

S3 Table Data containing presence/absence for the eight carnivores species recorded across the sampling stations in Nahuelbuta Mountain Area in southern Chile. (RAR)
