# Supplementary material for: Diurnal Human Activity and Introduced Species Affect Occurrence of Carnivores in a Human-Dominated Landscape
Source: PLoS One. 2015 Sep 14;10(9):e0137854. doi: 10.1371/journal.pone.0137854 (PMC4569270; doi:10.1371/journal.pone.0137854)
Supplement: S1 Table — (DOCX) [file pone.0137854.s001.docx]

S1 Table. Correlation coefficients (top) and variance inflation factors (down) for covariates used in models of carnivore occurrence and detection probability.

| Correlation | Elevation | NF250 | NF500 | Prk | Rd250 | Rd500 | Ptch250 | Ptch500 |
| --- | --- | --- | --- | --- | --- | --- | --- | --- |
| Elv | 1 |  |  |  |  |  |  |  |
| NF250 | -0.258 | 1 |  |  |  |  |  |  |
| NF500 | -0.263 | 0.663 | 1 |  |  |  |  |  |
| Prk | -0.244 | -0.233 | -0.224 | 1 |  |  |  |  |
| Rd250 | 0.1 | -0.452 | -0.483 | 0.342 | 1 |  |  |  |
| Rd500 | -0.004 | -0.55 | -0.585 | 0.391 | 0.611 | 1 |  |  |
| Ptch250 | -0.48 | 0.06 | 0.06 | -0.028 | -0.076 | -0.067 | 1 |  |
| Ptch500 | 0.206 | -0.017 | -0.04 | -0.053 | 0.079 | 0.07 | 0.102 | 1 |

| Covariate/species | Kd | Dog | Lg | Cf | Df | Gf | Co | Sk |
| --- | --- | --- | --- | --- | --- | --- | --- | --- |
| NF.plot | 1.751 | 1.320 | 1.46 | 1.16 | 1.71 | 2.01 | 1.67 | 1.9 |
| Elv | 1.675 | 1.190 | 1.750 | 1.673 | 1.555 | 1.305 | 1.119 | 2.525 |
| NF250 | 1.559 | 1.260 | 1.422 | 1.253 | 1.135 | 1.041 | 1.56 | 2.74 |
| NF500 | 1.358 | 1.900 | 1.230 | 1.280 | 1.120 | 1.550 | 1.620 | 1.660 |
| Prk | 1.121 | 1.760 | 1.010 | 1.110 | 1.540 | 1.760 | 1.900 | 1.220 |
| Rd250 | 2.532 | 3.130 | 2.780 | 2.210 | 2.900 | 1.980 | 2.100 | 1.760 |
| Rd500 | 3.149 | 2.980 | 3.030 | 2.790 | 2.880 | 3.120 | 3.220 | 3.120 |
| Ptch250 | 1.458 | 1.870 | 1.210 | 1.400 | 1.560 | 1.870 | 2.012 | 1.320 |
| Ptch500 | 1.258 | 1.430 | 1.110 | 1.090 | 1.800 | 1.470 | 1.346 | 2.112 |
| Season | 1.141 | 1.920 | 1.090 | 1.430 | 1.670 | 1.491 | 1.674 | 1.231 |
| Und | 1.041 | 1.100 | 1.113 | 1.556 | 1.359 | 1.517 | 1.845 | 1.765 |
