# Supplementary material for: Diurnal Human Activity and Introduced Species Affect Occurrence of Carnivores in a Human-Dominated Landscape
Source: PLoS One. 2015 Sep 14;10(9):e0137854. doi: 10.1371/journal.pone.0137854 (PMC4569270; doi:10.1371/journal.pone.0137854)
Supplement: S2 Table — The occurrence of dogs was estimated from a null model. (DOCX) [file pone.0137854.s002.docx]

| S2 Table. Posterior model probabilities for the set of best-supported candidate models (i.e., with posterior probability >0.05) for the detection (p) probabilities of introduced dog in Nahuelbuta Mountain Area in central-south Chile. Dog's occurrence was estimated from a null model. | | | |
| --- | --- | --- | --- |
| Species | Day | Night | Posterior probability |
| Dog | ψ(.) *p*(Season+Und) | ψ(.) *p*(Season) | 0.37 |
|  | ψ(.) *p*(Und) | ψ(.) *p*(Season+Und) | 0.18 |
|  | ψ(.) *p*(Und) | ψ(.) *p*(Und) | 0.09 |
|  | ψ(.) *p*(.) | ψ(.) *p*(.) | 0.06 |
|  | ψ(.) *p*(Season) | ψ(.) *p*(Season) | 0.05 |
